# Comparing contribution of bony and cartilaginous endplate changes to intervertebral disc degeneration

**DOI:** 10.12669/pjms.40.7.8762

**Published:** 2024-08

**Authors:** Amber Salman, Asma Sajjad Khawaja, Kashif Baig, Uruj Zehra

**Affiliations:** 1 Amber Salman, MPhil. Department of Anatomy, University of Health Sciences, Lahore, Pakistan; 2 Asma Khwaja, MBBS. Department of Anatomy, University of Health Sciences, Lahore, Pakistan; 3 Kashif Biag, FCPS. Department of Pathology, Aziz Fatima Medical and Dental College, Faisalabad, Pakistan; 4 Uruj Zehra, PhD. Department of Anatomy, University of Health Sciences, Lahore, Pakistan

**Keywords:** Cartilaginous endplate, Bony endplate, Intervertebral disc degeneration, Osteophytes

## Abstract

**Objective:**

To compare the degenerative features of cartilaginous endplate with bony endplate in association with intervertebral degeneration in local population at radiographic, macroscopic and microscopic level in human motion segments.

**Methods:**

This cross-sectional descriptive study examined 59 lumbar spine motion segments from adult male cadavers at the Department of Anatomy, University of Health Sciences, Lahore, between May and September, 2022. Radiographic assessment observed bony endplate (BEP) for the presence of sclerosis & osteophytes and degeneration scores from 1-8 were assigned. Macroscopic assessment was done to evaluate BEP, cartilaginous endplate (CEP) and IVD, and scores ranged from 1to 28 for BEP, 1-4 for CEP and 1-64 for IVD were assigned. Microscopic assessment revealed degeneration scores of CEP ranged from 1-42 and 1-30 for IVD. Segments with BEP defects were also identified on radiographs & macroscopy.

**Results:**

Significant correlations were observed between the total degeneration scores of BEP with IVD and CEP scores (r=0.88 and r=0.909, respectively, p<0.001). Similarly, the total degeneration scores of the CEP is also significantly correlated with total IVD (r=0.86, p<0.001). Additionally, the samples with BEP defects were having higher IVD degeneration scores (p<0.001).

**Conclusion:**

This study, for the first time identifies that there exists a critical association of bony and cartilaginous endplate with intervertebral disc degeneration individually in the same tissue sections using multi-dimension assessment methods. Degeneration in any of the components of VEP is consonantly associated with IVD degeneration. The BEP & CEP, though, they are unique structures but are interlinked with each other structurally and functionally.

## INTRODUCTION

Global burden of disease study in 2015 revealed ‘spinal ailments’ as ‘a strain on the productive life’ which took a huge surge between 1990 and 2015 worldwide and affected all age groups.^1^ In the United States, low back pain (LBP) affects 10-33%, costing over $100 billion annually^2^ while Asian studies report 12-42% prevalence.^1^ In Pakistan, few small cohort studies on bankers and health professionals observed its prevalence between 52-68% which is quite alarming.^3^,^4^ Despite an 84% lifetime prevalence of chronic back pain, its exact cause remains elusive, notably, degenerative disc diseases involving discovertebral joint contribute prominently.^5^

All the components of discovertebral joint such as intervertebral discs, longitudinal ligaments, cartilaginous and bony endplate are linked to each other in such a way that degeneration of one element affects the other, but degrees of the changes are not uniform.^6^ Intervertebral disc is a fibrocartilaginous pad that is sandwiched between superior and inferior vertebral endplates. The two components of vertebral endplate, bony & cartilaginous, are morphologically distinct structures each having unique roles. The cartilaginous layer acts as a physical barrier between the blood vasculature of the bony vertebra and intervertebral discs and at the same time protects the nucleus from bulging into the vertebral body. It helps equalize the load between vertebral body and intervertebral discs and maintains the hydrostatic pressure. Any breach can compromise the biomechanics and immune privileged status of the intervertebral discs.^7^ Therefore, a critical association exists between the intervertebral discs and vertebral endplates. The complex link between vertebral endplate changes and disc degeneration has been demonstrated in many previous studies in which these vertebral endplate alterations including Modic changes, defects, sclerosis and calcification were identified to be associated with disc degeneration.^8^

However, bony and cartilaginous endplates were taken as a single unit in most of the studies due to the limitations of imaging diagnostic tools in clinical and research settings. The commonly reported vertebral endplate changes based on imaging emphasize frequently on bony endplates missing the changes of cartilaginous endplates. That is the most likely reason behind the conflicting association of vertebral endplate and disc degeneration.^9^ Keeping in view the functional importance of cartilaginous endplate it is plausible to assume that it is the cartilaginous endplate which is notorious and may be more influential leading to disc degeneration and pain.^8^ It is evident that cartilaginous endplate degeneration can be common, with age and degeneration composition of the cartilaginous endplate undergoes several changes which are difficult to identify in clinical and research settings. Being an avascular structure and compromised reparative ability the likelihood of regeneration and healing after damage is preposterous.^10^

It is crucial that features of cartilaginous endplate (CEP) should be distinguished from bony endplate (BEP) degenerative changes and explore their association with intervertebral disc (IVD) degeneration independently. This study was designed to compare the degenerative features of cartilaginous endplate with bony endplate in association with intervertebral degeneration in local population at radiographic, macroscopic and microscopic level in human motion segments.

## METHODS

A descriptive cross-sectional study was conducted in the Department of Anatomy University of Health Sciences, Lahore on 59 lumbar spine motion segments dissected out from 13 adult male cadavers of age range between 20-80 years. These cadavers were obtained from the Anatomy Department of Faisalabad Medical University between May and September 2022.Through naked eye and radiographic examination, the spine with any gross deformity and/or with evidence of any pyogenic or tuberculous lesions were excluded from the study.

### Ethical Approval

### It was obtained from Ethics Committee of University of Health Sciences, Lahore under letter no. UHS/REG-21/ERC/6602.

### Radiographic Assessment of Bony Endplates

**Fig.1:**
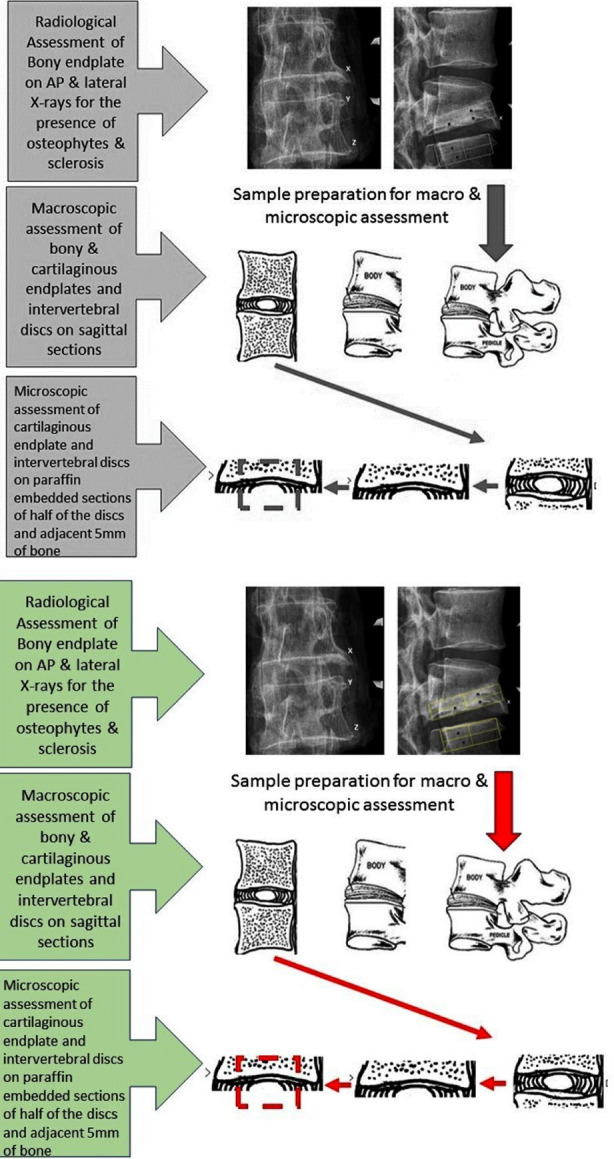
Schematic image showing all three modes of assessment for bony and cartilaginous and intervertebral disc degeneration.

The anteroposterior and lateral radiographs of the lumbar spine motion segments were taken in the radiology department of Madinah Teaching Hospital Faisalabad (X Ray machine model # D150 L) to observe the BEP sclerosis and osteophytes and based on these two radiological scores of degenerations ranging from one to eight was awarded using a standardized protocol^8^ (Fig.1). Along with that, presence of any defect was also observed. The BEP sclerosis was observed as a white opacity surrounding the upper and lower endplates in the samples, while osteophytes presented as bony projections hanging from the vertebral margins in both anteroposterior and lateral views.

### Macroscopic Assessment of Bony & Cartilaginous Endplate and Intervertebral Discs

Each sample was then divided mid-sagittal into left and right halves and both components of vertebral endplate along with intervertebral disc were observed (Fig 1), IVD was further observed by making the transverse section and individual scores to each of the three assigned based on standardized macroscopic scoring system published before.^8^ It ranged from 1to 28 for BEP,1-4 for CEP and 1-64 for IVD. Briefly the scores were assigned on the basis of defects in the CEP & BEP. Focal defects were identified and scored as interruptions in the continuity of the BEP, which progressed to larger fractures in some samples. In IVD the consistency of nucleus pulposus was observed and scored on the basis of its appearance from a gel-like to fibrosed state, along with the presence of fissures and brown discoloration. While in the annulus fibrosus, the signs of lamellar disorganization, fissures, and calcification were observed and scored.

### Microscopic Assessment of Cartilaginous Endplate and Intervertebral Discs

Histological sections were obtained from half of the IVD and adjacent 5mm piece of bony and cartilaginous endplates (Fig.1). The H&E stained sections were observed for different histological changes and scores were assigned based on standardized protocol ranged from 1-42 for CEP and 1-30 for IVD.^8^ The CEP changes were scored on the basis of variation in its thickness, the presence of cracks, calcification, alteration in cellular distribution, matrix disorganization and separation from the vertebral endplate. The changes in the IVD such as the presence of fissures, granular alterations, cellular proliferation or decreased cellularity were observed and scored.

### Statistical Analysis

Data was entered and analyzed using the latest version of Statistical Packages for Social Sciences (SPSS 21.0). Mean ± SD were calculated for quantitative variables. The correlation between individual scores of BEP, CEP and IVD based on radiological, macroscopic and microscopic scores were assessed by Pearson correlation coefficient. The statistical difference between IVD scores of degenerations in samples with and without BEP defect was analyzed using independent sample t test. P-value < 0.05 was considered to indicate statistical significance.

## RESULTS

### Bony Endplate Features & Scoring on Radiographs

Almost 58% of the motion segments were well-aligned with no signs of sclerosis or osteophytes on X-rays. However, in remaining individuals, sclerosis and osteophytes were prominently evident. Most samples exhibited uniform upper and lower endplates, but rounded BEP defects characteristic of Schmorl’s nodes were observed in 16% of the motion segments. The sclerosis scores ranged between 1-4 while its mean ± SD was 2.2 ± 0.83. The osteophytes scores ranged between 1-3 while its mean ± SD was 1.8 ± 0.69. The mean degeneration scores of BEP combining sclerosis and osteophyte for individual motion segment was 4.03 ± 1.1.

### Bony & Cartilaginous Endplate and Intervertebral Disc Features & Scoring on Macroscopy

**Fig.2:**
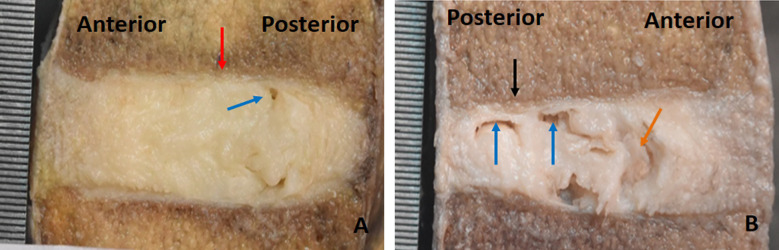
Sagittal sections showing gross features of intervertebral discs and endplate degeneration . A: Non generated samples with a uniform cartilaginous endplate( red arrow) with small fissure in IVD (blue arrow). B: Degenerated sample with fissures in the nucleus (blue arrow), resorption of CEP (black arrow) and brown discoloration within the intervertebral disc (brown arrow).

The CEP was recognized as a thin white line that separates the IVD from the vertebra. In nearly all samples, the thickness of the CEP was irregular, and in some instances, minor fractures were observed, resulting in protrusion into the vertebral body. Focal defects were prevalent in 36% of the BEP (Fig.2). The nucleus pulposus within the IVD exhibited different consistencies, spanning from a normal gel-like state to a fibrous one. Small, multiple fissures were observed within the IVD, with occasional extension into the CEP. Brown discoloration was noted in only a few discs. In the annulus fibrosus, samples exhibited signs of lamellar disorganization, fissures, and calcification (Fig.2). The individual degeneration scores of BEP, CEP & IVD ranged between 5-25, 3-7 & 15-43 respectively, while their mean ±SD were, 13.9±5.6, 3.61± 1.1 & 26.13±7.8 respectively.

### Cartilaginous Endplate and Intervertebral Disc Features & Scoring on Microscopy

H&E sections revealed the CEP lying between intervertebral disc tissue and BEP. The lateral few millimeters of each specimen were generally devoid of any cartilaginous endplate, therefore, the fibers of the outer annulus fibers inserted directly into the subchondral bone as Sharpey’s fibers. Varied cellular distribution within the CEP was observed, with increased cellularity and disorganized matrix in degenerated specimens. Cracks or defects, hypo-cellularity, and microfractures were detected in the CEP. The junction between CEP and BEP showed loose connections and natural cracks.

**Fig.3:**
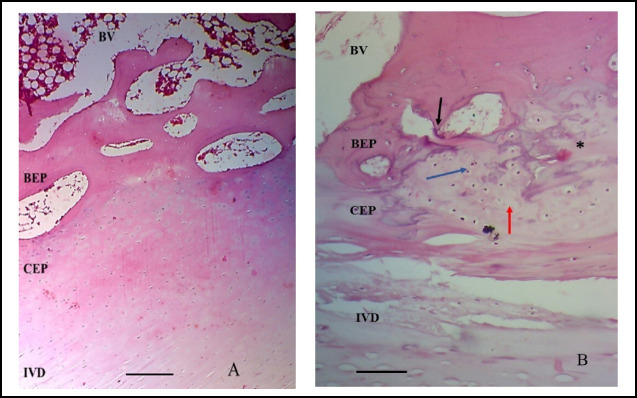
Tissue sections on H&E staining showing microscopic features of intervertebral discs and endplates degeneration. A: Non degenerated sample where BEP, CEP & IVD are well preserved. B: Showing fractured BEP (black arrows), hypercellularity (blue arrow), hypocellularity (red arrow) and dis-organized matrix (asterisks) in CEP. Note the fissures in IVD lamellae 200X, scale bar = 50µm. BEP: Bony endplate; CEP: Cartilaginous endplate; IVD: Intervertebral disc.

Microfractures were noted in the BEP, sometimes filled with CEP. Calcification areas within the CEP were observed where the BEP protruded. The intervertebral disc displayed an organized matrix with fewer cells, while nucleus pulposus fibers exhibited a wavy appearance. Cellular proliferation, cluster formation, and granular matrix were noted in degenerated discs. Strong junction between disc and CEP was observed due to fiber insertion from inner annulus fibrosus and nucleus pulposus. Fiber bundles from nucleus pulposus and inner annulus penetrated the CEP, with outer annulus fibers parallel to it (Fig.3). The individual degeneration scores of CEP & IVD ranged between 10-36 & 4-22 & respectively, while their mean ±SD were 20.9± 7.7 & 11.1±4.1 respectively.

### Correlation between Endplate (Bony & Cartilaginous) & Intervertebral Disc Degeneration

The BEP scores from radiological and macroscopic observations were added to get the maximum score of 36 (8+28=36), while CEP and IVD scores from macroscopic and microscopic observation were cumulated to get the maximum scores of 46 (CEP=4+42=46) and 94 (IVD= 64+30=94). Total scores obtained in the samples of BEP, CEP and IVD were ranged between 1-36, 1-46 and 1-94 respectively.

The total degeneration scores of the BEP was significantly correlated (r=0.88, p<0.001) with the total degeneration scores of the IVD and CEP (r=0.909, p<0.001). Similarly, the total degeneration score of the CEP was also significantly correlated with total IVD (r=0.86, p<0.001). (Table-I) Samples with BEP defects observed on radiology and macroscopically had significantly greater (p<0.001) IVD scores (mean 46.9± 8.5) as compared to the group without it (mean 29.4±5.5).

**Table-I:** Showing Pearson correlation coefficient between total degeneration scores of the BEP, CEP and IVD with each other. BEP: bony endplate, IVD: intervertebral disc and CEP: cartilaginous endplate.

	BEP	CEP	IVD
BEP	1	r=0.909, p<0.001	r=0.88, p<0.001
CEP	r=0.909, p<0.001	1	r=0.86, p<0.001
IVD	r=0.88, p<0.001	r=0.86, p<0.001	1

## DISCUSSION

This study comprehensively analyzed the impacts of bony and cartilaginous endplates on intervertebral disc degeneration in human motion segments. Employing a multidimensional approach, including radiographic, macroscopic, and microscopic assessments, it revealed significant insights into the individual and synergistic roles of these components in disc degeneration, enhancing the understanding of their intricate relationships. These findings suggest that both bony and cartilaginous endplates equally influence intervertebral disc degeneration as observed by other study^11^, emphasizing their unified function as a crucial barrier regulating nutrient transmission, load distribution, and maintaining the blood-nucleus barrier’s integrity.^12^

Radiological scores for BEP degeneration, considering sclerosis and osteophyte, increased with severity, in line with prior study.^8^ Macroscopically, as degeneration progressed, increase in focal defects, resorption, and fractures of the bony endplate were observed, reflected by rising macroscopic scores, as reported in previous study.^8^ Our study combined these scores to present a novel method to express overall BEP degeneration that also followed the similar pattern. This increase in scores could be due to the hypertrophic responses of bone, either due to cartilage loss or biomechanical stress^13^ or the degenerative changes may trigger abnormal collagen-to-bone matrix replacement, spatial reorganization, hypertrophy, and neovascularization, leading to sclerosis.^14^

Similarly, increase in macroscopic scores for CEP degeneration was observed as it appeared diffused with varied thickness and protruding into adjoining vertebra. While it’s microscopic scores of degeneration were increased with increasing disorganization, fissuring and altered cellular distribution. Our study is innovative in presenting cumulative scores of CEP degeneration, derived from both macroscopic and microscopic observations, to indicate the severity of degeneration, which also increased proportionally. These findings are consistent with prior studies which linked these structural changes, such as fissures and defects to microtrauma and prolonged mechanical strain, triggering modifications in the matrix and weakening of the CEP.^14^,^15^

The macroscopic scores of IVD degeneration increased with the appearance of fissures, focal defects and brown discoloration in nucleus pulposus and annulus fibrosus, whereas its high microscopic scores of degeneration were due to the increased in the presence of chondrocyte clusters, amorphous and granular materials owing to ongoing degeneration. Our study combined both macroscopic and microscopic scores of IVD degeneration that also increased with severity. Similar findings are reported in prior studies where these changes mirrored the tissue remodeling in degenerated IVDs which could influence the balance between load transmission and nutrient exchange^12^,^16^. Correlation analysis revealed significant connections between BEP, CEP, and IVD degeneration scores, consistent with previous research emphasizing their pivotal role in spinal health.^14^,^17^-^19^

CEP degenerative changes may trigger abnormal collagen-to-bone matrix replacement, spatial reorganization, hypertrophy, and neovascularization, leading to sclerosis.^14^ This impairs nutrition and potentially allows immunocyte infiltration, previously blocked by NP blood barrier. Inflammatory cells, cytokines, and pathogens alter the intradiscal environment, accelerating endplate degeneration.^15^ Inflammatory mediators induce ROS release, cartilage osteogenic differentiation, calcification, and cellular senescence, impacting the adjacent disc as well.^16^,^17^

The novel feature of this study is that it evaluated the comparative effects of BEP and CEP on IVD degeneration in the same samples, suggesting a shared pathophysiology where changes in one component can influence the other. Consistent with previous findings, our results indicated that degenerated CEP may compromise nutrition, potentially facilitating immunocyte infiltration previously impeded by the NP blood barrier. This infiltration can alter the intradiscal environment, accelerating endplate degeneration. Inflammatory cells, cytokines, and pathogens induce ROS release, cartilage osteogenic differentiation, calcification, and cellular senescence, impacting the adjacent disc as well^20^,^21^. These findings are consistent with previous studies highlighting the importance of the CEP in maintaining the health of the IVD and its susceptibility to degeneration.^12^,^14^,^16^,^18^

This study represents the first documented account within the Pakistani population. Despite the unique demographic characteristics, the results remain in alignment with previous investigations, featuring the crucial roles of bony and cartilaginous endplates in maintaining intervertebral disc health. Evaluation of the degeneration scores of CEP & BEP in the same samples and determining the association with IVD degeneration is another novel feature of this study.

### Strengths & Limitations of the Study

The current study conducted a comprehensive analysis of bony and cartilaginous endplate characteristics and their association with intervertebral disc degeneration across various assessment levels for the first time. Robust correlations were observed between degeneration scores of BEP, CEP, and IVD, underscoring their interdependence. But like any other study few limitations do exist a more useful comparison of CEP and BEP changes could have been obtained if clinical (history of low back pain) & demographic data (age, weight life style and diet) of the individual was present especially with low back pain but due to the nature of the study (cadaveric) the relevant details could not be obtained.

### Future Recommendations

Further research in this field has the potential to facilitate the development of targeted interventions aimed at mitigating or preventing spinal degeneration, considering the unique contributions of these structural components.

## CONCLUSIONS

The findings collectively highlight the intricate relationships and clinical relevance of BEP and CEP attributes in intervertebral disc health, offering valuable insights into spinal degeneration mechanisms.

### Authors’ Contribution:

**AS:** Sample collection, all experimental work, recorded all observation, compiled results and wrote first draft of manuscript.

**AK:** Helped in experimental work and in observation.

**KB:** Helped in sample collection and experimental work.

**UZ:** Conceived the idea, designed the study, interpreted the data and helped in compilation of results. Revised and finalized the manuscript. Overall supervision and facilitated the study.

All the authors read and approved the final manuscript.
